# The Nightingale study: rationale, study design and baseline characteristics of a prospective cohort study on shift work and breast cancer risk among nurses

**DOI:** 10.1186/1471-2407-14-47

**Published:** 2014-01-29

**Authors:** Anouk Pijpe, Pauline Slottje, Cres van Pelt, Floor Stehmann, Hans Kromhout, Flora E van Leeuwen, Roel CH Vermeulen, Matti A Rookus

**Affiliations:** 1 Netherlands Cancer Institute, Department of Epidemiology, Plesmanlaan 121, 1066 CX Amsterdam, the Netherlands; 2 Institute for Risk Assessment Sciences, Division of Environmental Epidemiology, Jenalaan 18d, 3584 CK Utrecht, Utrecht, the Netherlands

**Keywords:** Shift work, Night work, Occupational exposures, Breast cancer, Chronic disease, Nurses

## Abstract

**Background:**

Evidence for the carcinogenicity of shift work in humans is limited because of significant heterogeneity of the results, thus more in-depth research in needed. The Nightingale Study is a nationwide prospective cohort study on occupational exposures and risks of chronic diseases among female nurses and focuses on the potential association between shift work and risk of breast cancer. The study design, methods, and baseline characteristics of the cohort are described.

**Methods/Design:**

The source population for the cohort comprised 18 to 65 year old women who were registered as having completed training to be a nurse in the nationwide register for healthcare professionals in the Netherlands. Eligible women were invited to complete a web-based questionnaire including full job history, a detailed section on all domains of shift work (shift system, cumulative exposure, and shift intensity) and potential confounding factors, and an informed consent form for linkage with national (disease) registries. Women were also asked to donate toenail clippings as a source of DNA for genetic analyses. Between October 6, 2011 and February 1, 2012, 31% of the 192,931 women who were invited to participate completed the questionnaire, yielding a sample size of 59,947 cohort members. The mean age of the participants was 46.9 year (standard deviation 11.0 years). Toenail clippings were provided by 23,439 participants (39%).

**Discussion:**

Results from the Nightingale Study will contribute to the scientific evidence of potential shift work-related health risks among nurses and will help develop preventive measures and policy aimed at reducing these risks.

## Background

Nurses experience potential exposure to a wide variety of chemical, biological, physical, and psychosocial exposures in the course of their work. An association which has been extensively debated over the last decades is shift work and its potential hazardous effect on breast cancer risk. Shift work has also been related to numerous other health problems, among which are cardiovascular disease, metabolic disorders, digestive troubles, fatigue, depression, anxiety and sleep problems [1,2]. Exposure to light-at-night was first suggested to contribute to the increased incidence of breast cancer around three decades ago [3,4]. Based on a literature overview, the International Agency for Research on Cancer (IARC) concluded in 2007 that in animals there was ‘sufficient experimental evidence’ for the carcinogenicity of light during the daily dark period but ‘limited evidence’ for the carcinogenicity of shift work that involves night work in humans, resulting in an overall classification that ‘shift work that involves circadian disruption as ‘probable carcinogenic to humans (group 2A)’ [5,6].

There are several hypotheses about the biological mechanisms underlying the potential health effects of shift work. They include the suppression of melatonin secretion by light at night, circadian rhythm disruption (phase shift and desynchronization of clock genes), depression of immune function, decreased production of vitamin D, unhealthy lifestyle changes, and long-term sleep disruption and deprivation [7]. These effects could lead to direct and indirect changes in hormonal, immunological, and metabolic parameters that may be related to the development of adverse health effects such as cancer. Melatonin has been shown to have indirect effects on the neuroendocrine reproductive axis and acts as a selective estrogen receptor modulator and a selective estrogen enzyme modulator [8]. Because of the effects of melatonin on estrogen levels and the role of estrogens in the development of breast cancer, the most common malignancy among women worldwide [9], research on potential carcinogenic effects of shift work has focused on breast cancer risk.

So far, 18 epidemiological papers have been published on the association between shift work and the risk of breast cancer (excluding studies among flight attendants) [10-27]. Recent reviews of this literature, by Bonde et al. [28], Kamdar et al. [29], Jia et al. [30], and Ijaz et al. [31] have provided little more clarity on the potential association between shift work and breast cancer risk in humans than what was known at the time of the IARC report. Human evidence lags behind because of significant heterogeneity of the results, most likely due to variations in study design, the lack of standardized definition and assessment of shift work, the retrospective character of the majority of the included studies, and lack or incomplete adjustment for potentially important confounding factors and effect modifiers like reproductive factors, lifestyle but also genetics and chronotype.

The term “shift work” has been widely used and generally includes any arrangement of daily working hours other than the standard daylight hours (7/8 am – 5/6 pm) [6]. Night work, which can be conducted according to a permanent or a rotating schedule, is thought to have the most disruptive effects on the circadian rhythm [32]. In 2010, night work was undertaken by 19% of European workers; 23% among men and 14% among women [33]. In this report, a night shift is defined as having to work for at least two hours between 10 pm and 5 am. With such a high prevalence of night work and its potential health effects, a large part of the workforce may be at increased risk of several chronic diseases. More rigorous epidemiological research is needed to understand the specific risks associated with shift work involving night work and the underlying biological mechanisms, and to provide more specific and evidence-based recommendations on the prevention of diseases related to shift work. As a stepping stone for future studies, an IARC working group has identified three major domains of shift work that should be captured in future studies: shift system, cumulative exposure, and shift intensity [34].

Here we present the rationale, design and methods of the Nightingale Study, a large Dutch prospective cohort study targeted at the investigation of associations between occupational exposures and risk of chronic diseases among female nurses with a focus on the assessment of the association between shift work and breast cancer risk. We hypothesize that an association between shift work and breast cancer risk may be attributed to specific domains and aspects of shift work and that individual factors like polymorphisms in certain circadian genes and chronotype may modify the association between shift work and breast cancer. The Nightingale Study was amongst others set up to meet the recommendations of more in-depth research on the potential health effects of shift work. The study covers more details concerning shift systems than previous studies. In this paper, we also present baseline characteristics of our cohort and compare our study population to those of similar cohorts (i.e. the Nurses’ Health Study I and II).

## Methods/Design

### Design and study population

In 2010, the Netherlands Cancer Institute (NKI) and the Institute of Risk Assessment Sciences (IRAS) of the Utrecht University, initiated the here described Nightingale Study. The Nightingale Study is a prospective cohort study aimed at the investigation of associations between occupational exposures and risk of chronic diseases. The primary aim is to study the potential association between shift work and risk of breast cancer. Other hormone-related cancers as well as other diseases such as cardiovascular and neurodegenerative diseases and their associations with nurses’ occupational and lifestyle exposures will also be investigated prospectively. Approval of the study procedures was obtained from the Institutional Review Board of the NKI. Eligible women were invited to complete a web-based questionnaire and an informed consent form (see sections on informed consent form and questionnaire for details). In addition, women were asked to donate toenail clippings (i.e. clippings of at least three nails) as a source of DNA for future analyses of genetic polymorphisms that may modify the associations between shift work and disease risks.

The nationwide register for healthcare professionals in the Netherlands (BIG-register) gave us permission to use the registry to contact all female (ex-)nurses. The BIG-register is based on individuals who obtained a relevant diploma, i.e. a nursing degree in our study, and who are then able to use the legally protected professional title as long as they fulfill requirements for regular training [35]. The BIG-register includes women who are currently employed as a nurse as well as women who changed careers and those who retired. Addresses and vital stats are kept up to date by automated linkage with the Municipal Personal Records Database. The BIG-register has an estimated inclusion rate of at least 95% among those who obtained a nursing degree. The source population for the Nightingale Study cohort comprised of 193,029 18 to 65 year old female BIG-registered nurses with a residential address in the Netherlands who met these inclusion criteria on July 28, 2011. The recruitment of participants for the Nightingale Study took place between October 6, 2011 and February 1, 2012. Of the selected women, 98 died between July 28 and October 6, 2011. Thus, in total, 192,931 women were eligible and invited to participate in the Nightingale Study.

### Pilot study

Prior to the main launch, we conducted a pilot study in which we investigated participation rates using two different data collection strategies: an online-only and a mixed-mode strategy (i.e. offering a web-based questionnaire at the initial invitation and a paper questionnaire along with the reminder letter), and the effect of a reminder letter. Four groups of 200 women each were randomly selected from the registry: 1) online-only, 18–39 years, 2) online-only, 40–59 years, 3) mixed-mode, 18–39 years, 4) mixed-mode, 40–59 years. Groups 1 and 2 received an invitation letter containing a username and a password to complete the study questionnaire online. Upon no response, a reminder letter, again containing a username and password, was sent after four weeks. Groups 3 and 4 received an invitation letter containing a username and a password together with the option to request, through a reply form, a paper-based version of the questionnaire. Upon no response, a reminder letter, containing the login codes but also a paper-based questionnaire, was sent after four weeks. The participation rates were 14%, 14%, 11%, and 22% for groups 1, 2, 3, and 4, respectively. The overall participation rate was 16%: 9% after the initial invitation and 7% after the reminder. The participation rate of both strategies was similar, although adding a paper-based questionnaire along with the reminder led to more responders in the older age group (participation rate 22%), even though a similar proportion of this group versus group 3 responded online (62% and 52% in the younger and older age groups, respectively, p = 0.604).

An evaluation survey among the non-responders in the pilot study resulted in several recommendations for improving our study materials, e.g. adaptation of the order of some items, improvement of phrasing and layout.

### E-cohort study

The result of the pilot study was one of the reasons to opt for the online-only strategy in the main launch, which is less time and money consuming. Other reasons were that in an online procedure data can be checked during completion (i.e. participants are directed automatically to applicable questions and they are notified of potential errors, e.g. having entered text in a numeric field) which results in higher quality of the data, no need for data entry and less data cleaning afterwards. We designed the online system to enable participants to save what they already completed and log off to log in again later to continue questionnaire completion. To ensure an adequate level of protection of the data (i.e. to prevent other individuals from accessing the participants’ data by using the login codes only) we implemented a verification system at the login site (i.e. ask zip code and date of birth after having paused). Upon completion, participants could save their informed consent form and answers in the questionnaire for their own purposes. One of the unique features of the online questionnaire system was a lifeline-graph (i.e. a line from birth to date of questionnaire completion) on which life events were depicted in the order of time during questionnaire completion as a memory aid. Examples of items that were depicted on the lifeline-graph were jobs and births of children.

### Recruitment

Just before the start of recruitment we launched a nationwide mass media campaign to publicize the study (i.e. we distributed a press release which resulted in articles in at least 10 newspapers and magazines, three interviews on national radio and an item in a primetime television news program). Furthermore, the study was actively supported and promoted by the Dutch Nurses’ Association (V&VN) and similar nursing organizations, associations, and magazines. Our study website (http://www.nightingale-studie.nl) was primarily developed as the gateway to the study questionnaire but was also designed to increase the participation rate and to provide background information on the why and how of the Nightingale Study to women who were invited to participate in the study and to the general public.

To guarantee the anonymity of registered individuals, the BIG-register forwarded our invitation letter, including a username (study ID) and password, to participate in the Nightingale Study to eligible women by regular mail. The BIG-register added a separate letter including the name and address of the individual and was signed by the head of the BIG-register to promote participation. The BIG-register kept a file with the link between the study IDs and the names and addresses; this file was destroyed after the recruitment period had ended. The study was presented as a study on health among nurses, covering occupational history, lifestyle, and environment. The invitation study pack consisted of the letter from the BIG-register, our invitation letter, a full color information leaflet including contact information for inquiries, a step-by-step plan on how to participate, a mini zip lock bag for toenail clippings, and a reply envelope (free of charge). Upon no response, a reminder letter, again through the BIG-register, was sent after five weeks. Both the invitation and reminder letter contained an URL link and the study ID and password to access the web-based questionnaire and informed consent form through the study website (i.e. http://www.nightingale-studie.nl). Women who wanted to participate in the study on genetic susceptibility were asked to put their toenail clippings in the mini zip lock bag, labelled with a barcode sticker with their study ID, and return the sample in the reply envelope. Women who wanted to decline participation could do so through the study website (i.e. decline form), through e-mail or telephone. The response rate was defined as the percentage of invitations that resulted in a response. A response could be a decline, complete participation (i.e. informed consent and at least half of the questionnaire completed including the section on occupational history and exposures and main confounding factors), or incomplete participation (i.e. informed consent yet less than half of the questionnaire completed). The participation rate was defined as the percentage of invitations that resulted in complete participation.

A number of eligible women did not receive the invitation letter because it was lost during the mailing process (number unknown; national estimate of lost mail is about 1%), because the mail was returned undeliverable (n = 960, <1%) or because women were lost to follow-up by the BIG-register due to emigration or unsuccessful linkage to Municipal Personal Records Database due to missing personal data (estimated n ≈ 3,000, ≈1.5%). Therefore, we developed a self-registration system on the study website to give these women the opportunity to sign up for the study themselves. The self-registration system was designed in a way that only women with a BIG-register number or women who had a nursing degree could sign up.

### Response and participation rates

Table 1 shows the response and participation rates at baseline of women eligible for the Nightingale Study (N = 192,931). The response rate was 40% (N = 79,932), including two percent declining participation and seven percent who started participation but did not complete the study questionnaire. For 960 women the mail was returned undeliverable. The overall participation rate was 31% and was somewhat higher among older women than among younger women (36% and 29% for 40–65 year olds and 18–40 year olds, respectively, P < 0.001). The participation rate before the reminder was 17%. Among the 59,947 participants, 23,439 (39%) returned toenail clippings.

**Table 1:** Response and participation rate in the Nightingale Study

	**N (%)**
**Eligible and invited**	192,931
Response received (responders)	76,932 (40%)
No response received	115,039 (60%)
Lost to follow-up (mail returned undeliverable)	960 (<1%)
**Responders**	
Declined participation	3,526 (2%)
Questionnaire completed^a^ (participants)	59,947 (31%)^b^
Incomplete questionnaire^c^	13,459 (7%)
**Participants**	
Questionnaire only	36,508 (61%)
Questionnaire and toenail sample	23,439 (39%)

^*a*^*Includes 4,889 women who filled in at least half of the questionnaire (i.e. the most important part on occupational and other risk factors) but did not complete the entire questionnaire.*

^*b*^*Includes 179 women who participated through the self-registration system.*

^*c*^*Women who did completed less than half of the questionnaire.*

### Informed consent form

To register for the main study women had to complete a web-based informed consent form prior to filling in the questionnaire. This included consent for 1) prospective follow-up on disease occurrence, death, and cause of death through record linkage with national (disease) registries like the Netherlands Cancer Registry (NCR) [36], the National Pathology Database (PALGA) [37], Statistics Netherlands (CBS), and the Central Bureau for Genealogy (CBG), 2) medical record review, 3) the use of toenail clippings for DNA- analyses (e.g. breast cancer susceptibility, radiation sensitivity, and clock genes) if they had returned those, 4) follow-up questionnaire invitation, and 5) (inter)national data pooling (anonymous). On the informed consent form, participants completed their personal information, indicated if they wanted to receive the yearly study newsletter through e-mail, and signed the form electronically. After having signed the informed consent form and before they started filling out the study questionnaire, participants were asked if they also wanted to participate in a substudy on the use of mobile phones and health [38].

### Study questionnaire

The questionnaire was developed based on our previous experience with breast cancer risk factor and occupational exposure questionnaires, and adapted and improved to the Nightingale Study setting and population after extensive pre-testing of in particular the shift work section as described below. The questionnaire was designed to cover a variety of exposures on the job and during private life with a primary focus on risk factors for cancer, cardiovascular and neurodegenerative diseases and potential confounding factors. Items that were included in the questionnaire are listed in Table 2.

**Table 2:** Topics and items included in the Nightingale Study baseline questionnaire

**Topics**	**Items**
Socio-demographics	Date of birth, birth country of participant and her parents, marital status, current employment status
Reproductive history	Ever pregnant, pregnancies of at least 24 weeks (for each birth: date, gender, vital status at birth, duration of pregnancy in weeks and breastfeeding in months), number of pregnancies less than 24 weeks, infertility, age at menarche, age at menopause (no menstruation in the last 12 months and reason it stopped)
Education	Nursing and other degrees, and for each degree year of graduation
Occupational history	For each job conducted for at least six month: job type (caregiver, nurse (sector specified) or other (type and sector specified)), start and stop year, hours per week, physical load (sedentary, standing/walking, heavy)
Shift work	- Total number of years working night shifts during educational period (start and stop year)
	- For each job listed: ever/never conducted early morning shifts, evening shifts, night shifts and sleep shifts^a^ for at least six months
	- By job and shift type: number of shifts per month, number of shifts in a row, start and stop time of shift, rotation type (forward or backward rotating, variable, permanent), number of years (start and stop year), shifts on voluntary basis. Additional item for sleep shifts: proportion worked and slept. If women indicated that shift characteristics differed within a job, women were asked to complete these items for each period
	- For the most recent night work period the following items were reported: sleeping habits between 2 successive nights worked (hours, difficulty with falling and staying asleep, use of medication or other substances to sleep, light and sound circumstances at in the bedroom), time spent outdoors between 2 successive nights worked, light circumstances at work during the biological night, diet, timing of warm meal, regularity of eating and sleeping, activity after the last night worked, method of switching back to normal day-night rhythm, shift work adaptability compared to peers.
Occupational exposures	For each job listed: ever/never worked with X-ray examinations, fluoroscopic examinations, radiotherapy, MRI, artificial optical radiation, ultrasound equipment, dielectric heating, and/or industrial sewing machines, or near (i.e. within 5 meters) product/person detection gates, transmission installations, subway/train tracks, high-voltage network like power lines, and/or radar installations
Lifestyle	Current height, body weight (birth weight, current weight, weight at age 18, weight at ages 20–29, 30–39, 40–49, 50–59, 60–65), physical activity (walking/cycling and sport activity before age 18, sports activity at ages 20–29, 30–39, 40–49, 50–59, 60–65, and walking, cycling, sporting, gardening, do-it-yourself, housekeeping in the past summer and winter), time spend outdoors in the past summer and winter, smoking (ever, current, age at start, total duration, and number of cigarettes), alcohol consumption (ever, age at start, number of units in the past year and at ages 20–29, 30–39, 40–49, 50–59, 60–65), and dietary pattern and regularity of eating and sleeping
Lifetime mobile phone use	Past and current mobile phone use, hands free use, preferred side of the head during mobile phone use supplemented by information on current and prospective use obtained from the network operators. In addition, past and current cordless phone use [38].
Residential history	Lifetime residential history (i.e. place of residence in the Netherlands), for the assessment of environmental exposures.
Current sleeping habits	MOS sleep scale, light and sound circumstances in the bedroom, chronotype
‘Night shifts’ that is not work-related	Period-specific information on disruptions of sleep because of personal circumstances (e.g. young children, social engagements). Items include start and stopping ages, mean number of nights per week disrupted, and number of hours awake during those nights
Current health and Medical history	General health assessment (1 item of SF12) and items on headaches (Headache impact test, ID-migraine), hearing, tinnitus; cancer, benign lesions, cardiovascular diseases, neurodegenerative diseases, and metabolic disorders (ever diagnosed and age at diagnosis), and surgeries
Prescribed drugs	Period-specific information on use of hormonal contraceptives, hormone replacement therapy, hormones for IVF treatment, and on prescribed drugs like aspirin, medication for heart diseases, sleeping pills, diabetes medications, antidepressants, immunomodulators, and medications for Parkinson’s’ disease and asthma
Use of dietary supplements	Items on multivitamins, vitamin D, and calcium: ever/never, age at first use, age at last use, number of years use in total. For melatonin period-specific information was reported: start- en stopping ages and whether the use was daily or only during periods of circadian disruption
Diagnostic and therapeutic radiation exposures	Number of fluoroscopies, chest X-rays, coronary angiogram/angioplasty, CT-scans, diagnostics involving radioisotopes, and mammograms for age categories <20, 20–30, and after age 30; radiotherapy (age and location)
Family history of diseases	For mother, father, brothers, sisters, and children: diabetes, Parkinson’s’ disease, dementia, stroke, myocardial infarct, asthma, hay fever, and cancer of the lung, breast, prostate, ovary, uterus, colon/rectum. For grandmothers and aunts: breast cancer, ovarian cancer, and uterus cancer

^*a*^*Early morning shift: starting between 5.00 am and 6.59 am; evening shift: having worked at least one hour after 7.00 pm and with the shift ending no later than midnight; night shift: having worked at least one hour between midnight and 5.00 am; sleep shift (including weekend and on call shifts): having slept at work and woken up to work whenever necessary.*

Lifetime occupational history (i.e. history of jobs conducted for at least 6 months) was asked backwards (i.e. we first asked about the current job and then about previous jobs). During the completion of the job history, jobs that the participant had already filled in were listed on top of the page to remind the participant which jobs she had already reported. Shift work and other occupational exposures were linked to individual jobs listed in the occupational history section.

The shift work section of the questionnaire was developed to capture the major domains of shift work as listed by the IARC working group. These include shift system (e.g. start and stop time of shift, rotating or permanent, and speed and direction of a rotating system), years on a particular non-day shift schedule and cumulative exposure to the shift system over the subject’s working life, and shift intensity (i.e. time off between successive work days on the shift schedule) [34]. The shift work section of the questionnaire was improved after pre-testing through 1) evaluation in a focus group among three nurses of the NKI and 2) completion of the questionnaire by volunteers including nurses (n = 20 in three rounds). The minimum frequency and duration for all shift work types was having worked at least one shift (i.e. one evening or night or early morning) per month for at least 6 months. Participants could also indicate if they had worked day shifts only or if they had worked shifts for less than 6 months or less than 1 shift per month. Details on which shift work variables were included in our questionnaire are given in Table 2. For all these variables we collected calendar year-specific information to calculate cumulative exposure to certain shift systems over a subject’s working life.

### Baseline characteristics of participants

The mean age of participants at cohort entry was 46.9 years (SD 11.0 years). Participants were on average 2.3 years older than those who did not respond to our invitation (n = 115,039, mean 44.6 years, SD 11.0 years, P < 0.001). The median self-reported duration of questionnaire completion was 60 minutes (IQR 45–90). Table 3 shows the baseline socio-demographic characteristics of the Nightingale Study participants. The majority of participants was of Dutch origin (96%), married or in a de facto relationship (80%), and employed (86%). Educational level was equally distributed between a medium (i.e. intermediate vocational education, 53%) and a high level (i.e. ≥college, 47%) of education. In total, 2,009 participants (3.6%) reported to have had breast cancer (includes both in situ and invasive breast cancer).

**Table 3:** Baseline socio-demographic characteristics of 59,947 participants

**Characteristic**	**N (%)**^**a**^
**Age at cohort entry**	
18–25 year	3,070 (5%)
26–35 year	8,351 (14%)
36–45 year	12,858 (21%)
46–55 year	22,139 (37%)
56–65 year	13,529 (23%)
**Country of birth**	
Netherlands	57,701 (96%)
Born elsewhere	2,136 (4%)
**Top-5 country of birth when born elsewhere:**^**b**^	
Former Dutch East Indies (Indonesia)	313 (15%)
Germany	269 (13%)
Suriname	266 (13%)
Belgium	196 (9%)
Antilles (Aruba, Bonaire, Curacao, St. Marten, St. Eustatius)	143 (7%)
**Married or living together as married**	48,258 (81%)
**Highest educational qualification**	
Intermediate vocational education/community college)	31,593 (53%)
Higher vocational/professional education/college/university of applied science)	22,131 (37%)
University or higher	6,223 (10%)
**Employment status (most applicable)**	
Employed/self-employed	51,401 (86%)
Home duties/caregiver/volunteer	3,348 (6%)
Unemployed (but able to work)	438 (<1%)
Retired	2,539 (4%)
Unable to work	1,663 (3%)
Studying	456 (<1%)
**Monthly income estimate**^**c**^	
Low	799 (2%)
Medium	43,197 (81%)
High	9,117 (17%)

^a^Numbers do not always add up to 100% due to missing values.

^b^The Dutch East Indies, Suriname and the Antilles were all Dutch colonies.

^c^based on linkage individual zip codes with income data of Statistics Netherlands; for each individual the allocated income is the average income in December 2008 in the individuals’ zipcode area (PC6); in December 2008 the cut off value for having a low income was based on 40% of people with lowest income) and that for a high income was based on 20% of people with highest income. Source: CBS Kerncijfers postcodegebieden 2008–2019, http://www.cbc.nl.

### Night work

**Figure 1:**
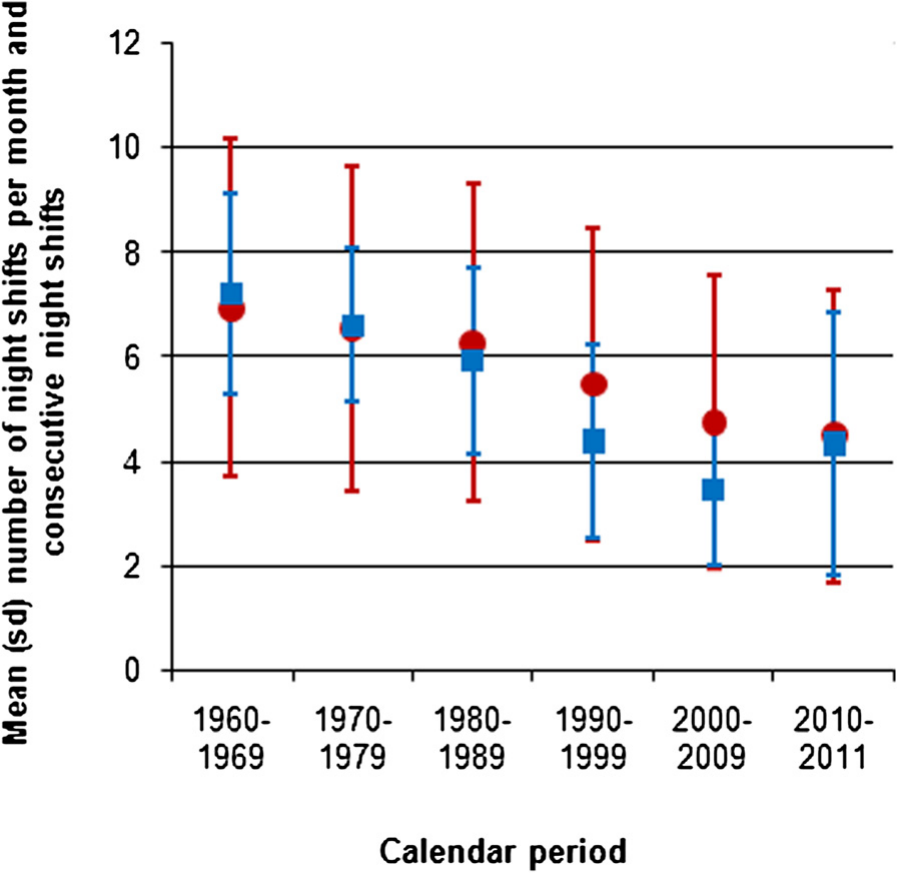
**Mean number of night shifts per month and number of consecutive night shifts by calendar period (1960–2011).** Legend: red dots indicate the number of nights worked per month; blue squares indicate the number of consecutive nights worked.

Eighty percent of participants indicated to have ever worked night shifts (i.e. ≥1 night/month for at least six months; not including educational period). Of those, 65% provided detailed information on different aspects of their night shift work. There were small but statistically significant differences in age and educational level between those who did provide detailed information on night shift work and those who did not (mean age difference 1.1 year, p < 0.01; proportion providing details was 64%, 66%, and 67% for low, medium and high level of education, respectively, p < 0.01)*.* Among 31,265 participants who indicated to have ever worked nights and who provided period-specific information, the mean lifetime duration of night shift work was 11.9 years (SD 8.4 years); 20% had worked at least 1 night per month for 20 years or more. The mean cumulative number of nights worked lifetime was 782.9 (SD 772.1). Almost one-third (27%) of the night shift workers had worked 1,000 nights or more during their entire life. Over all calendar years, the mean number of nights per month was 5.4 (SD 2.9). Figure 1 shows that the mean number of nights worked per month decreased from 6.9 in the sixties to 4.5 nowadays. Similarly, the mean number of consecutive nights worked decreased from 7.2 in the sixties to 4.3 nowadays. We observed that there was a downward trend with increasing age as well (data not shown). This decrease may reflect changes in policy and regulation of shift work but also labor market changes (e.g. changes in the proportion of women in part-time jobs). The proportion of women working in shift work schedules that are variable (and thus not fixed) was 77% among those working nights at cohort entry; this proportion did not change much over the years (data not shown). In contrast, the proportion of women working night shifts on a voluntary basis (i.e. able to self-schedule, indicate preferences, and swap night shifts with colleagues) has increased from 16% in the sixties to 43% nowadays. The proportions of participants who indicated to have ever worked evening shifts and sleep shifts were 89% and 21%, respectively.

Self-reported chronotype was distributed as follows: 12% of participants were a definite morning type, 23% were a probable morning type, 25% indicated to have no preference, 22% were a probable evening type, 11% were a definite evening type, and the rest did not know (1%) or the item was missing (7%).

### Other occupational exposures

**Figure 2:**
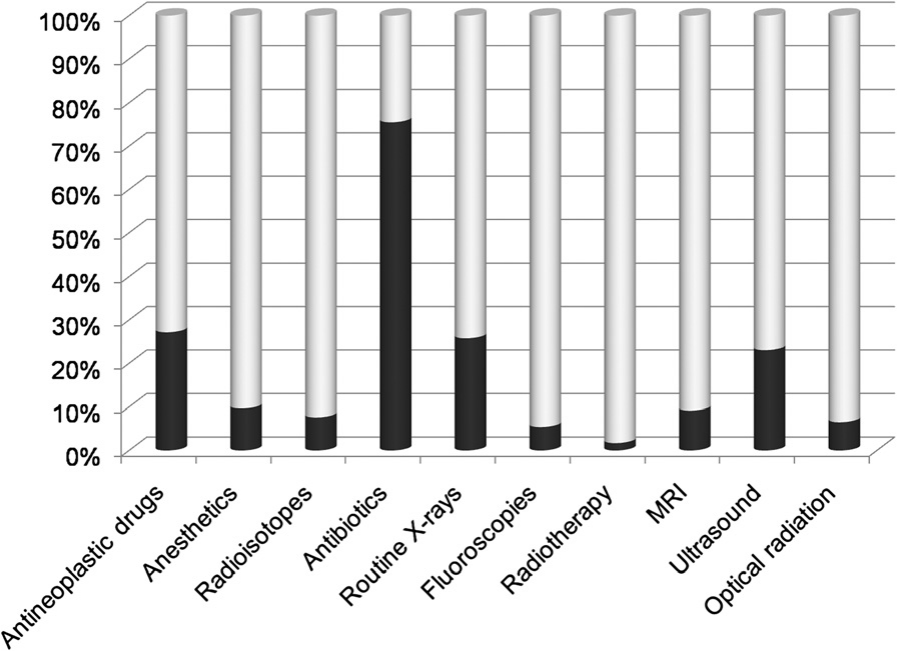
**Frequency of self-reported other occupational exposures among 59,947 Nightingale Study participants.** Legend: Dark part of bar indicates the proportion of participants who answered ‘yes’ to the following question ‘Have you ever worked with …… regularly for at least 6 months?

Besides shift work, the study questionnaire also covered other potential occupational exposures. Figure 2 depicts the frequencies of self-reported occupational exposures at baseline. The majority (75%) indicated to have ever worked with antibiotics for at least six months. Approximately one-quarter of participants had worked with antineoplastic drugs (27%), routine X-rays (26%), or ultrasound (23%). The frequencies of the other occupational exposures ranged from 2% for radiotherapy to 10% for anaesthetic gases.

### Statistical analyses

Information on (breast) cancer diagnoses will be obtained prospectively from the NCR, which has cancer diagnoses complete until two years prior to linkage, and PALGA, which has cancer diagnosis complete until two weeks prior to linkage. Information on tumor subtypes will be retrieved from the NCR. Vital status and primary and secondary causes of death will be obtained from CBS and CBG. We will closely examine the characteristics of those who reported to have never been engaged in shift work while indicating to have worked as a nurse. Shift work conducted during the years of nursing school was not collected in detail and only the total number of years of having conducted shift work during the educational period was ascertained. Statistical methods standard for the analysis of prospective cohort studies will be used. For example, the association between shift work and the risk of (breast) cancer will be evaluated using Cox proportional hazards regression with age as the time scale. For the incident breast cancer analyses, breast cancer cases prevalent at baseline will be excluded. Based on age-specific breast cancer risks [39], we expect 589 incident cases of breast cancer among participants unaffected at baseline in the first 5 years of follow-up. With a probability of disease at baseline of 1%, we will have 80% power to detect a relative risk of 1.36 for the highest versus the lowest level of exposure with five levels of exposure (e.g. duration of night work in five categories, see Table 4).

**Table 4:** Night shift work characteristics of 59,947 Nightingale Study participants

**Night shift work characteristic**	**N (%)**
Ever/never^a^	
Never	11,799 (20%)
Ever	48,050 (80%)
**Total number of years worked on night shifts**^**b**^	
1–4 years	5,695 (19%)
5–9 years	9,349 (31%)
10–14 years	6,103 (20%)
15–19 years	3,612 (12%)
20–24 years	2,668 (9%)
25–29 years	1,676 (6%)
≥30 years	1,395 (5%)
**Cumulative lifetime number of nights worked**^**b**^	
<250 nights	6,082 (23%)
250–499 nights	6,026 (23%)
500–749 nights	4,567 (17%)
750–999 nights	2,977 (11%)
≥1000 nights	7,093 (27%)
**Number of nights worked per month at cohort entry**^**c**^	
1 night per month	653 (7%)
2 nights per month	1,351 (14%)
3 nights per month	1,945 (21%)
4 nights per month	2,275 (24%)
5–7 nights per month	1,993 (20%)
>7 nights per month	1,223 (12%)
**Number of consecutive nights worked at cohort entry**^**c**^	
1 night in a row	625 (7%)
2 nights in a row	2,307 (25%)
3 nights in a row	3,092 (33%)
4 nights in a row	2,209 (24%)
5 nights in a row	637 (7%)
6 nights in a row	209 (2%)
7 nights in a row	262 (3%)
>7 nights in a row	20 (<1%)
**Type and direction of shift system at cohort entry**^**c**^	
Permanent	690 (7%)
Forward rotating	1,323 (14%)
Backward rotating	27 (<1%)
Variable	7,287 (77%)
Don’t know/missing	85 (1%)

^a^Ever night shift work was defined as having worked at least one hour between midnight and 5 am for at least one night per month.

^b^Among 31,265 participants who ever worked night shifts and provided detailed information.

^c^Among 9,889 participants who worked nights at cohort entry (i.e. in 2011).

In the present paper, basic descriptive statistics were used, focusing on night shifts. Categorical variables were reported as frequencies and continuous variables as the mean (standard deviation, SD) or median (interquartile range, IQR). Differences between groups were assessed with a chi-square or t-test.

## Discussion

### Main findings

To our knowledge, the Nightingale Study is the first prospective cohort study on shift work and breast cancer risk in which at baseline detailed data were collected on all domains of shift work, as defined in the international consensus paper by Stevens et al. [34]. Given the large size of the cohort (N = 59,947) and its wealth of data the cohort is well poised to investigate the possible associations between occupational risk factors, in particular shift work, and chronic diseases, in particular cancer. Moreover, an important feature of our study is the collection of toenail clippings for analyses on biological mechanisms involving the circadian clock (i.e. genetic effect modifiers). The Nightingale Study participants are heterogeneous in age and shift work history which enables us to study into great detail amongst others dose response relationships, combination variables of shift work domains (i.e. shift system, cumulative exposure, and shift intensity), and age and time lag/latency effects.

### Representativeness

The participation rate in our study was 31%. With regard to age and educational level, our study population seems to be a representative sample of the total population of nurses in the Netherlands: the difference in age between participants and those who did not respond to our invitation was small and the proportion with a higher versus a lower educational qualification in our study (i.e. 53% intermediate vocational education and 47% higher vocational education or higher, see Table 3) was slightly higher but similar to what was reported in 2009 by van der Windt et al. on the Dutch nursing population (i.e. 60% and 40%, respectively) [40]. The number of prevalent breast cancer cases (n = 2,009, 3%) was somewhat higher than expected based on the 10-year point-prevalence on January 1^st^, 2010 (0,9% [41]) indicating a possible overrepresentation of breast cancer cases. However, these prevalent cases prevalent at baseline will be excluded for the incident case analyses. Selection bias based on non-response is not an issue in prospective cohort studies because the exposure was assessed before the outcome of interest occurs.

### Comparison with other prospective cohort studies among female nurses

So far, 18 epidemiological papers have been published on the association between shift work and the risk of breast cancer (excluding studies among flight attendants because of co-linear cosmic radiation exposure) [10-27]. Among those, two were prospective cohort studies involving female nurses, i.e. the Nurses’ Health Study I and II (NHSI and NHSII) in the United States [21,22]. In Table 5, we present several characteristics of these two studies with regard to design and study populations for comparison with our study. We also depict the distribution of lifetime duration of working night shifts in our study population, categorized according to the classification as used in the NHS.

**Table 5:** Comparison of night shift work characteristics of the Nurses’ Health Study I and II to the Nightingale Study data

**Study (name, country)**	**Participation rate baseline questionnaire (%)**	**N baseline**	**Mean age (range) at baseline**	**Follow-up period and identification of incident breast cancer cases**	**Night shift work definition and data collection**	**Lifetime duration of having worked night shifts (in years) among women *unaffected *** **with breast cancer at baseline**		
						Years on rotating night shift	Nurses’ Health study	Nightingale Study data^a^
Schernhammer et al. 2001 [21] (Nurses’ Health Study I, USA)	Baseline questionnaire in 1976 had 71% response rate. 85% of those responded to the 6^th^ biennial-mailed questionnaire in 1988 which included an item on night shift work	1988: N=103,613 of which 85,197 answered shift work question of which 78,562 were unaffected with cancer	54.3 (43–67) years [43]	1988-1998; every two years, cohort members receive a follow-up questionnaire with questions about diseases and health-related topics; breast cancer confirmed through medical records	Ever having worked *rotating* nights shifts with at least *three* nights per month in addition to day or evening shifts in that month (answer in 8 prespecified categories: never, 1–2, 3–5, 6–9, 10–14, 15–19, 20–29, ≥30 *years*); unclear whether night shift work was updated in biennial questionnaires after 1988	Never	31,761 (40%)	10,480 (20%)
						Ever	46,801 (60%)	43,116 (80%)
						1–14 years	40,993 (88%)	20,440 (69%)
						15–29 years	4,426 (9%)	7,612 (26%)
						≥30 years	1,382 (3%)	1,312 (5%)
Schernhammer et al. 2006 [22] (Nurses’ Health Study II, USA)	24% (see http://www.channing.harvard.edu/nhs/?page_id=70)	1989: N=116,671; 116,087 (99.5%) answered night work items; 115,022 unaffected with cancer	34.3 (25–43) years [43]	1989-2001; every two years, cohort members receive a follow-up questionnaire with questions about diseases and health-related topics; breast cancer confirmed through medical records	Ever having worked *rotating* nights shifts with at least *three* nights per month in addition to day or evening shifts in that month (answer in 8 prespecified categories: never, 1–4, 5–9, 10–14, 15–19, ≥20 *months*). Shift work information was updated in 1991, 1993, 1997, and 2001. In the 2001 questionnaire the night shift work item included rotating night shifts as before *and permanent night shifts for 6 or more months*	Never	35,153 (31%)	10,480 (20%)
						Ever	78,063 (69%)	43,116 (80%)
						1–9 years	70,773 (91%)	14,569 (50%)
						10–19 years	6,759 (9%)	9,338 (32%)
						≥20 years	531 (<1%)	5,457 (18%)

^a^The definition of night work in the Nightingale Study was: ever having worked permanent or rotating night shifts with at least one night per month for at least six months.

In the NHSI, three mailings resulted in participation rate of 71% in 1976 among 30 to 55 year old female nurses [42]. Among those who completed the baseline study questionnaire, which did not include items on shift work, 85% completed the 1988 follow-up questionnaire containing a night shift work question [21]. In the NHSII, started in 1989, the participation rate was around 24% after a single mailing [22]. In both the NHSI and NHSII, data were collected by means of a pre-printed study questionnaire. The age differences between participants in the two cohorts (NHSI: mean age = 54.3 in 1988, NHSII: mean age = 34.3 in 1989) indicated that NHSI was primarily a postmenopausal cohort while NHSII included mostly premenopausal women [43]. In both the NHSI and NHSII, participants were classified as a night shift worker when they had worked rotating night shifts with at least three nights per month. Night shift work was conducted by 60% and 69% of the NHSI and NHSII women, respectively. The majority of NHS night shift workers (i.e. 88-91%) had worked night shifts for no more than 14 years.

The distribution of Nightingale Study participants over the categories of night shift work as defined in the Nurses’ Health Study I and II shows that the Nightingale cohort has relatively more ever night shift workers who worked also for a longer period of time in night shifts. The differences in night shift work duration between our study population and that of the NHSI and NHSII are likely due differences in the definition and threshold of night shift work used and the lack of data on permanent night shift work in the NHS. Moreover, the NHSI and NHSII were both conducted in another country, time period and included women with a different age range, although the age range of our study population covers the age range of NHSI and NHSII when taken together.

### Strengths and weaknesses

The main strength of this cohort study of nurses is its large study population with a wide range of (levels of) both occupational and non-occupational (i.e. lifestyle and environmental) exposure(s), together with the ability to link with several registries with nationwide coverage to prospectively follow the participants regarding disease occurrence and cause of death. Another strength is the ability to approach almost all women aged 18 to 65 ever trained as nurses in the Netherlands through the cooperation of the nationwide register of healthcare professionals. Furthermore, our baseline study questionnaire covered the major domains of shift work as defined by international consensus. The relatively low threshold for shift work (i.e. at least one night per month for six months) enables us to study many different levels of both intensity and duration of shift work and to conduct a comprehensive dose–response analysis. The inclusion of only one occupational group enables us to study other exposures specific to nurses into great detail, although some nurse-related exposures may be correlated. Nurses were chosen because of the high prevalence of shift work and the focus of the study on breast cancer risk. Finally, we obtained data on a wide range of other (potential) risk factors and confounders, and we collected toenail clippings for analyses on biological mechanisms involving the circadian clock (i.e. genetic effect modifiers). With regard to genetic differences, we will analyze the DNA subtracted from toenails for genetic polymorphisms in circadian genes and melatonin metabolism genes. Circadian genes have been linked to both breast cancer risk [44-49], shift work adaptation [50,51], and chronotype [52,53]. Diurnal preference (i.e. chronotype) in itself has been reported to predict tolerance to shift work [54] and to be related to melatonin level [55]. We hypothesize that individual factors like polymorphisms in certain circadian genes and chronotype may modify the association between shift work and breast cancer. There may be a natural selection of individuals with a good shift-work adaptability based on chronotype and/or genetic polymorphisms in circadian genes to do night shift work throughout their life while individuals who report intolerance for night work at some point quit; these two groups may differ in breast cancer risk. The first study to investigate effect modification by polymorphisms in circadian genes in the association between shift work and breast cancer risk was recently published by Monsees et al. [56] The study, which was conducted within the NHSII, observed that women homozygous for the minor allele (AA) of NPAS2 Ala394Thr with ≥24 months of shift work had a 2.83-times higher breast cancer risk compared to homozygous AA women with <24 months of shift work (95% CI 1.47-5.56). The observed multiplicative association with breast cancer risk per minor allel (A) was 0.65 (95% CI 0.51-0.82) among women with <24 months of shift work and 1.19 (95% CI 0.93-1.54) with ≥24 months. Two other studies observed mixed results [57,58].

Like in many observational studies, our data are in part based on self-reported information. The use of self-reported shift work data was inevitable because the shift work as reported by our study participants was conducted over many years and locations and employers generally did not archive schedules. Moreover, such schedules, which may be considered to be the golden standard, may even not reflect actual exposure because in many institutions nurses in practice often swap night shifts among themselves, often at the last minute. The prospective nature of our cohort study eliminates differential misclassification bias. Still, participants were asked to recall their lifetime shift work exposure at a mean age of 46.9 years, thus non-differential misclassification is likely. Whether non-differential misclassification leads to bias to the null depends on the extent of the misclassification. To our knowledge, no study on the accuracy of self-reported data on history of shift work has been conducted. To assess the reliability of self-reported shift work exposure in our study, we aim to include some of the shift work items from the baseline questionnaire in a follow-up questionnaire and compare both self-reports. A second limitation of our study is that about one-third of the participants who indicated to have conducted shift work did not complete the detailed section on various aspect of shift work. We assume that perhaps questionnaire-fatigue or complexity of recall caused several participants to skip the detailed section as there were no large differences in characteristics like age and educational level between those who did and those did not complete it (data not shown). For this group of participants who did not complete the detailed shift work section we are able to derive type and duration from the occupational history section (i.e. for each job participants indicated if they had worked certain shifts) and by applying multiple imputation based on those participants who completed the detailed section. Finally, the fact that all Dutch nurses have to work nights during the years of education excludes the possibility to form a reference group of never exposed women.

### Challenges, experiences and recommendations

The challenges encountered during the study are related to the study population and to the data collection procedures. For example, it was a challenge to include items on all domains of shift work as listed in the international consensus paper by Stevens et al. [34] and all potential confounding factors. Even among nurses there are large differences in types, frequencies and timing of shifts conducted and we needed to design the questionnaire to fit all potential scenarios. Our questionnaire became rather lengthy and we were criticized for that by the participants. We think that if we would have added more items on certain shift work related or other aspects, which we would have liked to do, for example on diet and stress, the questionnaire completion rate or data quality would have dropped significantly.

Besides involving eligible women during the pre-test and pilot study, we recently established an advisory group consisting of eight participants to incorporate the participants’ view on the design, procedures and follow-up of the study. We highly recommend to other researcher to involve potential participants and their perspectives before a study is initiated. The experience of the pre-test and pilot study has given us the opportunity to improve the approach of the study population and the wording and design of the questionnaire and other study materials. We underestimated the time needed to develop the online system and all the features we wanted to implement. But the time invested has proven to be worthwhile as the participants indicated to appreciate very much features like the lifeline-graph, the possibility to log off and log in again later, and the opportunity to download a PDF file of their informed consent form and questionnaire for their own use after completion.

Other investigators planning to conduct large online prospective occupational cohort studies may encounter similar challenges as those encountered during this study. Our expertise as well as the way we addressed certain challenges, described in this paper, will hopefully contribute to an optimal design and conduct of future studies on the potential health effects of shift work.

## Conclusion

With the current high prevalence and unavoidability of night shift work and its potential health effects, a large number of people worldwide may be at increased risk of several chronic diseases. More rigorous research is needed to understand the specific risks associated with various domains of shift work involving night work and the underlying biological mechanisms, and to provide more specific and evidence-based recommendations on the prevention of diseases related to shift work. The extensive set of data acquired in the Nightingale Study will result in detailed knowledge regarding the potential health effects of shift work and other occupational exposures in female nurses, particularly the effects of different domains of shift work, dose–response, and age at time of shift work. Previously conducted studies on the association between shift work and breast cancer risk have a number of methodological limitations which has contributed to the current gaps in knowledge on this topic. Compared to previous studies, the Nightingale Study has several strengths, listed above. Future plans include amongst others conducting single nucleotide polymorphism (SNP) analyses in a case-cohort study design and updating shift work and other risk factor data through follow-up questionnaires, and to study the effect of pre- and postdiagnostic shift work on breast cancer survival among breast cancer survivors.

### Collaborations

We welcome collaborative research with the Nightingale Study data. For more information, please visit the ‘for researchers’ section on our website http://www.nightingale-studie.nl/Pages/for-researchers/.

## Abbreviations

IARC: International Agency for Research on Cancer; NKI: Netherlands Cancer Institute; IRAS: Institute of Risk Assessment Sciences; BIG-register: nationwide register for healthcare professionals in the Netherlands; V&VN: Dutch Nurses’ Association; NCR: Netherlands Cancer Registry; PALGA: National Pathology Database; CBS: Statistics Netherlands; CBG: Central Bureau for Genealogy; SD: Standard deviation; IQR: Interquartile range; NHS: Nurses’ Health Study; SNP: Single nucleotide polymorphism.

## Competing interests

The authors declare that they have no competing interests.

## Authors’ contributions

AP wrote the manuscript. AP, PS, CvP, HK, FvL, RV, and MR contributed to the design of the study. AP and MR coordinated the study and were responsible for study logistics, patient recruitment, and data collection. AP, CvP, and MR conducted the analysis of the pilot study. AP conducted the analysis of the response and participation rates, and the analysis of the baseline socio-demographic characteristics. AP, FS, and MR conducted the analysis of the night shift work characteristics. AP, FvL, RV, and MR are principal investigators. HK, RV, and FvL were responsible for the funding of the study. All authors revised the manuscript critically for intellectual content and have given final approval of the final manuscript.

## Pre-publication history

The pre-publication history for this paper can be accessed here:

http://www.biomedcentral.com/1471-2407/14/47/prepub
